# Communication between HIV-infected children and their caregivers about HIV medicines: a cross-sectional study in Jinja district, Uganda

**DOI:** 10.7448/IAS.17.1.19012

**Published:** 2014-07-17

**Authors:** Phoebe Kajubi, Susan Whyte, Simon Muhumuza, David Kyaddondo, Anne R Katahoire

**Affiliations:** 1 Child Health and Development Centre, School of Medicine, College of Health Sciences, Makerere University, Kampala, Uganda; 2 Department of Anthropology, University of Copenhagen, Copenhagen, Denmark

**Keywords:** children with HIV, antiretroviral therapy, HIV disclosure, therapeutic communication, Uganda

## Abstract

**Introduction:**

Knowledge of antiretroviral therapy (ART) among children with HIV depends on open communication with them about their health and medicines. Guidelines assign responsibility for communication to children's home caregivers. Other research suggests that communication is poor and knowledge about ART is low among children on treatment in low-income countries. This study sought to describe communication about medicine for HIV in quantitative terms from the perspectives of both children and caregivers. Thereafter, it established the factors associated with this communication and with children's knowledge about their HIV medicines.

**Methods:**

We undertook a cross-sectional survey of a random sample of 394 children with HIV on treatment and their caregivers at nine health facilities in Jinja District, Uganda. We assessed reported frequency and content of communication regarding their medicines as well as knowledge of what the medicines were for. Logistic regression analysis was used to determine the factors associated with communication patterns and children's knowledge of HIV medicines.

**Results:**

Although 79.6% of the caregivers reported that they explained to the children about the medicines, only half (50.8%) of the children said they knew that they were taking medicines for HIV. Older children aged 15–17 years were less likely to communicate with a caregiver about the HIV medicines in the preceding month (OR 0.5, 95% CI 0.3–0.7, *p*=0.002). Children aged 11–14 years (OR 6.1, 95% CI 2.8–13.7, *p*<0.001) and 15–17 years (OR 12.6, 95% CI 4.6–34.3, *p*<0.001) were more likely to know they were taking medicines for HIV compared to the younger ones. The least common reported topic of discussion between children and caregivers was “what the medicines are for” while “the time to take medicines” was by far the most mentioned by children.

**Conclusions:**

Communication about, and knowledge of, HIV medicines among children with HIV is low. Young age (less than 15 years) was associated with more frequent communication. Caregivers should be supported to communicate diagnosis and treatment to children with HIV. Age-sensitive guidelines about the nature and content of communication should be developed.

## Introduction

Globally, an estimated 3.4 million children under 15 years were living with HIV in 2010 and more than 90% of these were in sub-Saharan Africa [1]. Children under 15 years constitute 13% of the estimated 1.2 million people living with HIV in Uganda. The number of children with HIV receiving Antiretroviral Therapy (ART) rose from 17,000 in 2008/2009 to 26,669 in 2011 [2]. While the introduction of ART has improved survival among children with HIV, evidence suggests that sustaining adherence remains a challenge [3–5]. Commonly cited barriers to adherence include factors related to the patient/caregiver/family (age of the child, misinformation about HIV status and other unique challenges faced by children with HIV and their caregivers), factors related to the drug/medication (the demanding nature of ART treatment) and those related to the health care system [6–9]. On the contrary, communication has been stated to play a very important role in children's compliance to medication [3–5]. A study on disclosure of HIV status and adherence to daily drug regimens among children with HIV in Uganda found that children's adherence motivation came from knowing their HIV sero status and understanding why they needed the drug [5]. Children's knowledge of what the medication is for and how to take it correctly is largely dependent on their caregivers and requires open communication with children about their health and medicines [5,10]. While literature highlights the importance of communicating with children about their HIV diagnosis and treatment as they grow older, so as to enable them to be responsible for their health and adherence to ART [11,12], there is no consensus on when this should be done.

International, regional and national guidelines recommend that children infected with HIV should be informed about their diagnosis and treatment [10,13–16]. However, there are different opinions concerning the age at which the process should start. Whereas the World Health Organization (WHO) recommends that children of school age (6–12 years) should be told their HIV status, the African Network for the Care of Children Affected by HIV/AIDS (ANECCA) suggests that the process of informing children can start as early as 5–7 years [14,16]. The Uganda National Antiretroviral Treatment Guidelines on the contrary recommend that the process should start as early as seven years [10]. Although the above guidelines do not specify the content of messages to be communicated to the infected children, they suggest that the communication must be done gradually and in a culturally sensitive manner, with the consent and participation of the child's parents or caregivers [14]. The guidelines further indicate that the communication should be in a simple language, based on the child's age and understanding [10]. The Uganda National Policy on HIV Counselling and Testing stipulates that in no case should the provider or parent/guardian lie to a child of any age about their diagnosis [13]. All these guidelines emphasize the importance of considering the maturity of the child, ascribe the responsibility of disclosure to the caregivers and highlight the supportive role of the health worker in the communication process.

Despite the presence of guidelines, studies indicate that caregivers often hesitate to explain to the children what the treatment is for [5,12,17–28]. In Uganda, a study on disclosure of HIV status to children aged 5–17 years revealed that only 29% of the caregivers had explained to their children about HIV infection and its relationship to their medication [5]. Another study revealed that 25% of caregivers were unwilling to disclose to the children that they were infected with HIV [27].

Previous research on communication about children's HIV diagnosis and treatment has focused mainly on health care providers’ and caregivers’ perspectives on whether to disclose and when and how to inform infected children about their HIV/AIDS diagnosis and treatment [12,17,18,22,29]. However, little is known about children's perspectives on communication and their knowledge of their medicines.

This study sought to describe communication about medicine for HIV in quantitative terms from the perspectives of both children and caregivers. Thereafter, it established the factors associated with this communication and with children's knowledge about their HIV medicines.

## Methods

### Study design

This was a cross-sectional survey conducted between September and December 2011. The survey employed quantitative methods of data collection, while qualitative methods were used in another part of the larger study. The survey was followed by an ethnographic study that involved follow-up of a purposively selected sampled number of children for one year in different social spaces that mainly included homes, treatment centres and post-test clubs. The ethnographic study adopted participant observation and in-depth interviews as the main methods of data collection to enable an in-depth exploration of children's family situations, communication practices regarding their health and medicines, their challenges and concerns and how best they can be supported to live on ART. The results from the follow-up arm of the study are reported elsewhere.

### Study setting

The survey was conducted in nine health facilities providing ART in Jinja District, Eastern Uganda, where the overall HIV prevalence was estimated at 5.8% [2]. The District has a population of more than 380,000 people, with 79.1% living in rural areas [30]. More than half (56%) of the district's total population is aged below 18, of which 11% are orphans [30]. Subsistence farming, fishing and trading are the main economic activities in the district. The district has a total of 69 health facilities comprising 49 Government (public), 17 NGO and 3 Institutional (Army, Police and Prisons) health units. At the time of the study, six Government and three private not-for-profit health facilities were providing ART for children.

### Sample size

To determine the proportion of children who knew they were taking medicines for HIV, we estimated the minimum sample size of 384 children at a 90% power and 95% confidence level [31]. It was assumed that the proportion of children with HIV who knew that they were taking medicines for HIV was not known.

### Participants and recruitment

Children with HIV attending HIV clinics in all the nine health facilities that were providing ART in the district and their caregivers were enrolled in the study. Children did not have to know their HIV diagnosis to be included in the study. Children were considered eligible if they were aged 8–17 years, registered in the health facilities, were on HIV medicines and were accompanied by their primary caregivers on the day of the interview. A primary caregiver was defined as a person who lived with the child (including but not limited to biological parents), participated in the child's daily care and was the most knowledgeable about the child's health and medicine taking [5,32]. Primary caregivers were identified and screened for eligibility with the help of the paediatric counsellors.

### Sampling and data collection

The number of children with HIV sampled from each facility were randomly selected using systematic sampling. The HIV health facility registers where the names of the children are recorded were used as the sampling frame. The sampling interval was determined by dividing the total population of the children with HIV on treatment in the facility with the number of children to be studied in each facility (N/n). After obtaining a random start from a table of random numbers, the interval was followed until the required number of children from each facility was obtained. Experienced research assistants conducted interviews with each selected child and caregiver using two separate semi-structured questionnaires.

### Measures

In this study, the term communication referred to the verbal exchange of information about HIV medicines between children and their caregivers. Children's communication practices (frequency and content of communication) with their caregivers regarding their medicines and their knowledge of what the medicines were listed on the questionnaires. Measures of communication included, but were not limited to issues of disclosure. Through face-to-face interviews, children were asked about the number of times they talked about their medicines with their caregivers in the month preceding the interview. They were further asked what topics they discussed most (content) about the medicines with their caregivers. Content topics included 1) when to take the medicines, 2) when to stop taking them, 3) how to take the medicines, 4) side effects and 5) what the medicines were for. Caregivers were asked about their communication with the children concerning the medicines based on the opportunities that presented themselves. They were asked 1) whether they explained to the children reasons for the repeated visits to the health facility, 2) whether they explained to the children about the medicines they were taking, 3) whether the children understood what the medicines were for, 4) whether the children had ever asked what the medicines were for and 5) when they would stop taking them. Regarding knowledge of HIV medicines, children were asked if they knew what the medicines they took daily were for. The question that captured children's knowledge of what they were told the medicines were for was close ended. It had pre-coded answers that included TB, sickle cell, malaria, HIV, they didn't explain anything and other (specify). In addition, the socio-demographic characteristics of the children were investigated. Responses were recorded on the questionnaire, which had multiple-choice possibilities with an option of “others (please specify)” in case a child or care giver mentioned a response not captured on the questionnaire.

### Data management and statistical analysis

Double data entry validation was performed in Epi-Info software (version 7.1.2; Centres for Disease Control and Prevention, Atlanta, GA). After internal consistency checks, the cleaned data set was exported to STATA version 10.0 (TX, USA) for analysis. Mean and standard deviation (SD) were used to summarize continuous data. Bivariate analysis was done to identify factors associated with children's knowledge of what the medicines were for and communication about medicines with a caregiver in the preceding month using odds ratios (OR) and their 95% confidence intervals (CI). Multivariable logistic regression was done to identify the independent factors associated with children's communication about the medicines and knowledge of what the medicines were for.

### Quality control

The nine ART health facilities were pre-visited to obtain updated sampling frames. Both the children's and caregivers’ questionnaires were pre-tested for duration, language, clarity and sequencing of the questions to ensure their validation and suitability. The questionnaires were developed in English, and forward and back translated from Lusoga and Luganda (the local languages commonly used in the study area) to ensure reliability and validity. The questionnaires were separately administered by experienced graduate research assistants either in the local languages of Lusoga and Luganda or in English, depending on the language best understood by the respondents. Prior to data collection, the research assistants were trained in data collection and in the administering of the questionnaires. Questionnaire data were checked for completeness and accuracy before leaving the health facilities. The first author closely supervised the research assistants during data collection.

### Ethical considerations

Ethical clearance was obtained from Makerere University College of Health Sciences, Higher Degrees, Research and Ethics Committee and the Uganda National Council for Science and Technology. Permission to conduct the study was also obtained from the relevant political, administrative and technical authorities at the district and health facility levels. Written informed consent was obtained from caregivers for their own and children's participation in the study after explaining the objectives and procedures of the study to them. Assent to participate in the study was obtained from the children. Interviews were held in a private and quiet environment within the health facility premises. Confidentiality was maintained by the use of a coding system. The researchers worked closely with specialized paediatric counsellors from each of the nine health facilities where children with HIV were receiving treatment throughout the study to offer support and specialized counselling services to children when the need arose.

During the consenting and assenting process, the purpose of the research was explained to the caregivers and their children as a study of children's understanding and communication about their health and medicines. Through the entire fieldwork, the researchers were aware of the sensitivity of the subject, the study and questions were designed in such a way that they did not directly ask about HIV/AIDS but children's health and medicines. Care was taken not to ask questions in any way that would suggest or alert the child to their HIV status if they did not yet know it. The only time the word “HIV” appeared on the children's questionnaire was when children were asked, “What were you told the medicines are for?” Even in this case, the researchers never read out the different options but only filled in the children's response in the questionnaire, since it was interviewer administered.

Furthermore, given the sensitive nature of the children's condition and status, the issue of medication on which the study focused was a good pivotal point since it involved a daily practice in which all the children engaged. The focus on medicine helped to open up for other subjects related to taking medicine.

Even in this case, the researchers never used the word “ARVs” but rather used the word medicines. All of this was done to avoid inadvertent disclosure.

## Results

### Socio-demographic characteristics of children

A total of 394 children with HIV were interviewed. Of these, 55.3% were females. The mean age was 12.1 (SD 2.7). Almost all (93%) were attending school. The majority (52.3%) of the children lived with caregivers other than their biological parents. Of the 267 (67.8%) children who were orphans, 144 (53.9%) and 123 (46.1%) were single and double orphans, respectively. Age groups 11–14 and 15–17 had more double orphaned children, 57 (46.3%) and 50 (40.7%) compared to those aged 8–10 years.

### Socio-demographic characteristics of caregivers of children with HIV

A total of 393 caregivers of children with HIV were interviewed. Of these, the majority (80.9%) was female. Their mean age was 40 (SD 11.4). More than three-quarters (78.4%) had been to school and about half (50.3%) had attained secondary education and above. The most common occupation (41.5%) was subsistence farming.

### Caregivers’ communication to children regarding their medicines

The majority of the caregivers (61.3%) reported that they did not explain to the children why they were taking them to the health facility or reasons for the repeated visits to the treatment centres: 313 (79.6%) reported that they explained to the children about the medicines and 288 (73.3%) said that their children understood what the medicines were for. Most (63.9%) reported that their children have never asked them what the medicines were for and 266 (68%) reported that their children have never asked them when they will stop taking medicines (Table 1).

**Table 1:** Caregivers’ communication to children regarding their medicines

Characteristic	Frequency (N=393)	Percentage (%)
Caregiver explained to the child why s/he was bringing him/her to the clinic		
Yes	152	38.7
No	241	61.3
Caregiver explained to children about their medicines		
Yes	313	79.6
No	80	20.4
Does the child understand what the medicines are for?		
Yes	288	73.3
No	105	26.7
Has the child asked what the medicines are for?		
Yes	142	36.1
No	251	63.9
Has the child asked when s/he will stop taking medicines?		
Yes	127	32.3
No	266	67.7

### Children's reports on frequency of communication regarding medicines

When asked the number of times they talked about the medicines with their caregivers in the past month, 97 (24.6%) children reported that they did not talk about their medicines with their caregivers at all, 186 (47.2%) reported 1–2 times, 46 (11.7%) reported 3–5 times, while 65 (16.5%) children reported having talked about the medicines more than five times.

### Child-related factors associated with frequency of communication regarding medicines

Through a stepwise backward elimination method, all variables that were thought to be associated with frequency of communication regarding HIV medicines between children with HIV and their caregivers (children's age, sex, orphan status, school status and primary caregiver) were considered for inclusion in the multivariable model (Table 2). Age of the children was the only variable retained in the model. Older children (15–17 years) were less likely to talk with their caregivers about their medicines (AOR 0.53, 95% CI 0.31–0.72, *p*=0.002).

**Table 2:** Crude odd ratios (COR) and Adjusted odds ratios (AOR) and their 95% CI for factors associated with frequency of communication regarding medicines

Variable	Talked about medicines	Did not talk about medicines	Crude OR (95% CI)	*p*	Adjusted OR (95% CI)	*p*
Sex (n=394)						
Male	134 (45.1)	42 (43.3)	0.9 (0.6–1.5)	0.754	–	–
Female	163 (54.9)	55 (56.7)				
Age group-years (n=394)						
8–10	110 (37.0)	25 (25.8)	1			
11–14	124 (41.8)	36 (37.1)	0.8 (0.4–1.4)	0.400	–	–
15–17	63 (21.2)	36 (37.1)	0.4 (0.2–0.7)	**0.002**	0.53 (0.31–0.72)	0.002*
Orphan status (n=267)						
Single orphan	100 (52.1)	44 (58.7)	1.3 (0.7–2.3)	0.332	–	–
Double orphan	92 (47.9)	31 (41.3)				
School status (n=394)						
Out of school	18 (6.1)	11 (11.3)	2.0 (0.8–4.5)	0.084	–	–
In school	279 (93.9)	86 (88.7)				
Primary caregiver (n=394)						
Biological parent	86 (29.0)	35 (36.1)	1.4 (0.8–2.3)	0.187	–	–
*Other	211 (71.0)	62 (63.9)				

* Other refers to caregivers children lived with who were not their biological parents.

### Children's reports on content of communication about medicines

Children were asked whether they talked with their caregivers about when to take their medicines, when to stop taking medicines, how to take medicines, side effects of the medicines and what the medicines were for. This was a close-ended question and children were given choices from which they could select an answer. Of the 394 children, 135 (34.3%) reported that they talked with their caregivers about when to take their medicines; 115 (29.2%) mentioned that they talked about when they could stop taking medicines; 60 (15.2%) mentioned about how to take the medicines while, 53 (13.5%) reported that they talked about side effects of the medicines. Only 31 (7.9%) reported that they had talked of what the medicines were for.

### Children's knowledge of what the medicines were for

Of the 394 children, 200 (50.8%) reported they were taking medicines for HIV. Of the 194 who did not report that they were taking medicines for HIV, 45 (11.4%) said they did not know why they were taking medicines. The other children mentioned that they had been told they were taking medicines for TB 32 (8.1%); sickle cell 18 (4.6%); malaria 29 (7.4%) and other diseases, 70 (17.7%) including flu, coughs, skin rashes, syphilis, headache, stomach pains, worms, and measles.

### Factors associated with children's knowledge of what the medicines were for

The socio-demographic characteristics of the children were compared with their reported knowledge of what the medicines were for. The characteristics that were associated with children's knowledge of what the medicines were for at bivariate level (*p*<0.05) included age of the child, orphan status and having an aunt as a primary caregiver. These were considered for inclusion in the multivariable model. Only one variable, the age of the child, was retained in the final model (Table 3).

**Table 3:** Factors associated with children's knowledge of HIV medicines

Variable	HIV	Other illnesses	Crude OR (95% CI)	*p*	Adjusted OR (95% CI)	*p*
Age group-years (n=394)						
8–10	21 (10.7)	114 (57.9)	1			
11–14	92 (46.7)	68 (34.5)	2.5 (4.0–14.3)	<0.001	6.1 (2.8–13.7)	0.000*
15–17	84 (42.6)	15 (7.6)	33.3 (14.3–100.0)	<0.001	12.6 (4.6–34.3)	0.000*
Orphan status (n=267)						
Single orphan	70 (45.8)	74 (64.9)	2.2 (1.3–3.7)	<0.001	–	–
Double orphan	83 (54.2)	40 (35.1)				
Primary caregiver (n=394)						
Mother	49 (24.9)	72 (36.6)	1.7 (1.1–2.7)	0.012	–	–
*Other	148 (75.1)	125 (63.4)				
Primary caregiver (n=394)						
Aunt	37 (18.8%)	21 (10.7%)	1.94 (1.05–3.59)	0.023	–	–
**Other	160 (81.2%)	176 (89.3%)				

* Other referred to primary caregivers that children lived with who were not their biological mothers.

** Other referred to primary caregivers that children lived with other than aunt.

## Discussion

In this study, we found that children reported generally low frequencies of communication with their caregivers about medicines. A quarter reported that they had not discussed their medicines with their caregivers at all in the past month and about half said that they had talked about them once or twice. Frequency of communication about medicines was significantly associated with age: older children reported less communication with caregivers than younger ones. Our finding corroborate other research on family functioning in the context of paediatric chronic conditions, which found that increasing child age, especially adolescence, is accompanied by attempts to achieve increasing levels of autonomy, often leading to less parent–child cohesion and poor communication [33]. Research has shown further that moving towards independence from their parents, adolescents typically want to make their own choices and have a sense of control over their lives [9].

“What the medicines are for” was the least commonly reported topic of talk, while “the time to take the medicines” was by far the most mentioned by children. Other studies [18,34,35], as well as our own findings from the qualitative part of our research referred to in another paper [36], show that communication with children in settings such as Uganda is often directive, rather than participatory, with few opportunities for questioning, discussion, and joint decision making. When and how to take the medicines were talked about with caregivers but the communication was often directive and one-way, with caregivers instructing and reminding the children, rather than discussing the bigger issues of diagnosis, prognosis and lifelong medication. Teenagers would be expected to know how to take the medicines and to remember themselves when to take them. So this type of directive communication is less relevant for them and may explain the lower frequency for the older age group.

Based on disclosure guidelines and the Uganda National Policy on HIV Counselling and Testing, all children in our study were in the age bracket where they should ideally have known what the medicines they were taking were for [10,13,14,16]. However, only half the children reported that the medicines were for HIV/AIDS. This level of knowledge was higher than that reported by Bikaako-Kajura and colleagues from another study in Uganda, but their sample was smaller and it included children as young as five years [5]. A review of studies of HIV status disclosure to children in resource-limited countries shows lowest levels in Africa, and lower levels than we found [37]. That review also discusses the methodological difficulties of comparing levels of disclosure.

Despite the differences in orphan status and relation to the caregiver, logistical regression analysis showed that only age of the child was significantly associated with knowing that the medicines were for HIV. The importance of age is consistent with the results of other research on paediatric disclosure, which found that age remains the strongest predictor of whether or not the child has been disclosed to [25,37–39]. It is interesting that age is inversely related to frequency of communication. Older children's knowledge of their diagnosis does not seem to be associated with continuing frequent talk about their medicines with their caregivers.

A major finding of our study is that there is a clear discrepancy between caregiver and child reports about communication, including reasons for taking medicines. More than three quarters of caregivers affirmed that they had explained to the children about the medicines and that the children understood what the medicines are for and yet only half the children said they knew that the medicines were for HIV. The discrepancy could be due to several factors. Children may have refrained from mentioning that the medicines were for HIV, having been told to keep the secret as reported by studies on disclosure [12,40,41]. These studies noted that caregivers of children living with HIV almost always impose secrecy on the child about his/her status and children usually comply. In addition, caregivers in our study may also have given an optimistic picture of communication.

Nearly two thirds of caregivers reported that children did not ask what the medicines were for. However, qualitative research revealed that many children did want to know more about why they were taking medicines [36]. Either caregivers ignored children's curiosity or children actually did not pose questions directly to their caregivers. In cultural contexts such as Uganda where communication between adults and children is constrained, our findings agree with others that there is a need to develop interventions that promote communication about illness and take into consideration the child's age and development, family variables and cultural factors that influence communication as well as concepts of illness and death [42]. Vaz and colleagues suggest that interventions should consider how communication takes place within families to assist families in communicating illness information to children [18]. Since age is such a decisive factor in communication and knowledge about the medicines, there is particular need for age-sensitive guidelines about the nature and content of communication.

Our findings indicate missed opportunities where caregivers could have explained to children their diagnosis or why they were taking medicines. These include telling children why they brought them to the treatment centres repeatedly. About a third of the caregivers reported that the children had asked what the medicines were for and when they could stop taking them. Such questions also provide obvious occasions for communication. Our findings are in agreement with previous research that demonstrated that even though children's questioning and curiosity should have provided caregivers with an opportunity to have a dialogue with their children concerning their diagnosis, this did not often occur [17].

One potential limitation in this study was that data were based on informant responses, which might be subject to self-reporting bias. Both children and their caregivers could have given socially desirable answers. However, the quantitative responses were substantiated by open-ended questions in the survey qualitative interviews and participant observation [36].

Despite these limitations, reports from both caregivers and children helped to uncover the communication gaps and challenges regarding children's medicines. The inclusion of other aspects of communication, such as frequency and topic, is useful in placing the concern with disclosure in the broader context of everyday exchanges about medicines.

## Conclusions

Children's communication with caregivers about HIV medicines is infrequent and focused on taking tablets. Knowledge about the purpose of the medicines among children with HIV is low. This study reveals that relying on caregivers alone to communicate messages on diagnosis and treatment to children with HIV may be insufficient. Our findings suggest the need for interventions that prepare and support caregivers for this task. Where caregivers have difficulty assuming the task, health workers in some cases may assume the responsibility of explaining to the children, after consultation with the caregivers.

The WHO guidelines recommend that disclosure happens around age 6 but this does not commonly occur, as our findings show [16]. Guidelines should suggest how information can be packaged for the different age groups and how it can be given in a reassuring way. They should include frequently asked questions and sensitize caregivers to cues that children want to know more.

The assumption made by caregivers that children understand what the medicines are for contrasts with the children's own reports and underscores the importance of on-going communication about ARVs between caregivers and the children. Talking more frequently about the reasons for taking medicines in a natural way as the occasion arises may diminish the apprehension about diagnosis. If it becomes a pattern to discuss various aspects of medicines regularly, it will be more likely that children's understanding will develop over time as they grow up.
